# Optimizing Collaborative Crowdsensing: A Graph Theoretical Approach to Team Recruitment and Fair Incentive Distribution

**DOI:** 10.3390/s24102983

**Published:** 2024-05-08

**Authors:** Hui Liu, Chuang Zhang, Xiaodong Chen, Weipeng Tai

**Affiliations:** 1 School of Computer Science and Technology, Anhui University of Technology, Ma’anshan 243032, China; liuhui@ahut.edu.cn (H.L.); zhangchuang_319@163.com (C.Z.);; 2 College of Economics and Management, Nanjing University of Aeronautics and Astronautics, Nanjing 211106, China; 3 Engineering Research Institute, Anhui University of Technology, Ma’anshan 243000, China

**Keywords:** collaborative crowdsensing, Gaussian mixture model, graph theory, Shapley value, fairness

## Abstract

Collaborative crowdsensing is a team collaboration model that harnesses the intelligence of a large network of participants, primarily applied in areas such as intelligent computing, federated learning, and blockchain. Unlike traditional crowdsensing, user recruitment in collaborative crowdsensing not only considers the individual capabilities of users but also emphasizes their collaborative abilities. In this context, this paper takes a unique approach by modeling user interactions as a graph, transforming the recruitment challenge into a graph theory problem. The methodology employs an enhanced Prim algorithm to identify optimal team members by finding the maximum spanning tree within the user interaction graph. After the recruitment, the collaborative crowdsensing explored in this paper presents a challenge of unfair incentives due to users engaging in free-riding behavior. To address these challenges, the paper introduces the MR-SVIM mechanism. Initially, the process begins with a Gaussian mixture model predicting the quality of users’ tasks, combined with historical reputation values to calculate their direct reputation. Subsequently, to assess users’ significance within the team, aggregation functions and the improved PageRank algorithm are employed for local and global influence evaluation, respectively. Indirect reputation is determined based on users’ importance and similarity with interacting peers. Considering the comprehensive reputation value derived from the combined assessment of direct and indirect reputations, and integrating the collaborative capabilities among users, we have formulated a feature function for contribution. This function is applied within an enhanced Shapley value method to assess the relative contributions of each user, achieving a more equitable distribution of earnings. Finally, experiments conducted on real datasets validate the fairness of this mechanism.

## 1. Introduction

Crowdsensing [1], as a collaborative work method, has garnered widespread attention and research. Building on this, researchers, emphasizing collaboration and interaction among task participants to enhance task efficiency and quality, have introduced the concept of collaborative crowdsensing [2].

The effective recruitment and motivation of users to participate in collaborative crowdsensing projects is a key concern. By researching and analyzing the user recruitment and incentive mechanisms within collaborative crowdsensing, we can gain a better understanding of the motivations and needs of users participating in these projects and develop more effective strategies accordingly.

User recruitment is a crucial aspect of collaborative crowdsensing, involving attracting suitable participants to join the collaborative crowdsensing system and effectively managing their participation behavior and contributions. Researchers are dedicated to developing recruitment strategies, user evaluation methods, and user trust models to improve the effectiveness and efficiency of user recruitment. Xiao et al. [3] proposed a greedy approximation algorithm to solve the problem of deadline-sensitive user recruitment in probabilistic collaborative mobile sensing, addressing the recruitment of multiple users to collaboratively execute a common task to ensure the expected completion time does not exceed the deadline, thereby improving task completion efficiency and mobile user participation. Yang et al. [4], focusing on path-oriented participant recruitment, proposed a route-oriented solution to maximize road coverage by selecting appropriate participants, enhancing the effectiveness and cost-efficiency of collaborative crowdsensing. Wang et al. [5] found that participants are attracted by task labels and influenced by colleagues’ or friends’ intentions; hence, they addressed the issue of predicting the number of potential workers in spatial crowdsensing tasks and provided corresponding game models and algorithms, enhancing task management and efficiency. Yang et al. [6], aiming for maximized total benefits from recruited participants for sensing tasks within a given budget, designed a group recruitment algorithm based on genetic algorithms to maximize group sensing capabilities, considering three types of characteristics (coverage, reputation, and activeness), thus improving task execution efficiency and quality. Azzam et al. [7], for recruiting participants for given continuous sensing tasks, proposed a stability-based continuous sensing approach for group recruitment systems, addressing participant recruitment challenges and providing better information quality and cost-effectiveness.

The incentive mechanism is a critical component in collaborative crowdsensing, influencing participants’ enthusiasm and the effectiveness of task execution. Current studies mainly focus on designing fair and effective incentive mechanisms, such as reward-based, reputation-based, and competition-based systems, to stimulate participants’ motivation and creativity. For instance, CHAI et al. [8] utilized the self-interest of crowdsensing users by setting basic remuneration and providing additional rewards based on individual contributions. Rui et al. [9], based on repeated games, proposed a reputation-value-based incentive model to motivate rational workers to complete tasks with high quality and to impose corresponding penalties on malicious workers. Rokicki et al. [10], the first to study team-based crowdsensing competitions, conducted systematic research on the effects of collaboration and imbalanced reward distribution on the quantity and quality of annotations. They proposed a team-based crowdsensing competition method that uses competition and teamwork to improve cost-effectiveness and reduce crowdsensing costs. Han et al. [11], in the participatory sensing project BudBurst, introduced game incentives. This project aims to study the impact of climate change on plant growth by encouraging users to observe plants and record results.

In traditional crowdsensing user recruitment, current strategies mainly focus on users with optimal individual capabilities, yet often overlook their potential for collaboration. Additionally, most existing research on incentive mechanisms in collaborative crowdsensing is based on auction models from game theory. Because auction mechanisms require users to bid before completing tasks, in practice, most users do not know how to reasonably assess the costs of completing tasks and are reluctant to make frequent bids, thereby reducing their willingness to participate in tasks. Furthermore, in multi-person collaborative scenarios, there may be instances of some members free riding [12], or not honestly contributing their resources and labor, leading to issues of unfair profit distribution.

To address the aforementioned issues, this paper proposes an efficient recruitment strategy and incentive method. The recruitment strategy adopts a team-based approach, focusing not only on the individual abilities of team members but also on their collaborative capabilities. Based on this, we construct a user interaction graph and transform the team recruitment problem into solving the maximum spanning tree problem under multiple constraints in graph theory. We then employ an improved Prim algorithm to solve and select the team members, aiming to maximize the team’s overall effectiveness. This recruitment mechanism is named the IPA-TRM (Improved Prim algorithm-based Team Recruitment Mechanism).

Reputation value [13] is a quantifiable indicator based on a user’s historical performance and evaluations from other users, reflecting the user’s trustworthiness and overall performance over a certain period. This article integrates the reputation mechanism with a reward system, devising a comprehensive incentive mechanism. This mechanism utilizes members’ reputations to indirectly reflect their contributions within the team and applies it to the allocation of team benefits in collaborative crowdsensing. This incentive method is named the MR-SVIM (Member Reputation and Shapley Value-based Incentive Mechanism).

The contributions of this article are as follows: (1) It considers both the collaborative abilities and individual capabilities of users during user recruitment. (2) It introduces a Team Recruitment Mechanism based on the IPA-TRM, transforming the team recruitment problem into a maximum spanning tree problem in graph theory. It proposes an improved Prim’s algorithm to compute the optimal solution and demonstrates the effectiveness of the algorithm. (3) It presents a profit distribution method based on the MR-SVIM, offering an effective solution to address profit distribution issues in collaborative crowdsensing projects; this is effort is pioneering in similar literature. (4) By utilizing the PageRank algorithm and Shapley value method, it considers each member’s contributions and negative factors within the team, ensuring fairness and rationality in profit distribution, and thereby adhering to the principle of equitable distribution. (5) It leverages the transparency and tamper-resistant nature of blockchain technology to store member reputations on the blockchain. Utilizing smart contracts, it automatically computes member earnings, enhancing data security and credibility.

## 2. System Architecture

### 2.1. System Model

**Figure 1:**
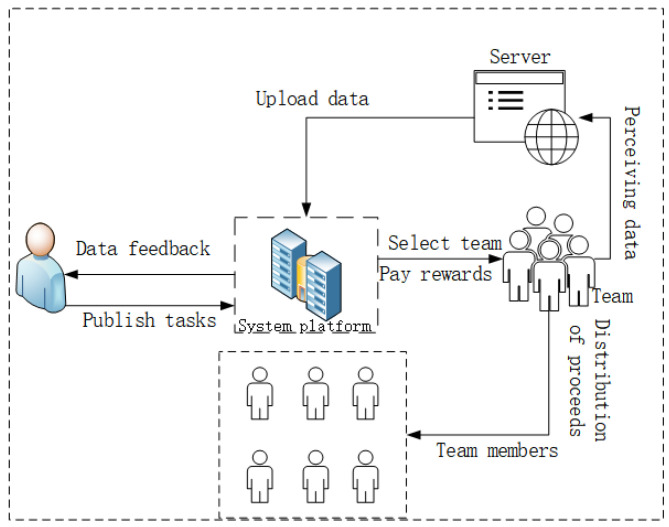
System model diagram.

The system model proposed in this section considers a real scenario [14], wherein task requesters post tasks on the platform. The platform selects suitable teams from a large pool of teams to execute the tasks and eventually delivers the data back to the requester. In the collaborative crowdsensing process, how to recruit users to form teams and distribute rewards to incentivize members is an essential consideration. The system model is illustrated in Figure 1.

Firstly, task requesters post their tasks on the platform. The platform, based on the task’s characteristics and requirements, selects members with strong collaborative abilities to form a team and complete the task. After the team members execute the task, they upload the data to the platform. The platform processes the data and provides the results to the task requester. Simultaneously, based on the task’s completion status, the platform pays the team’s reward. Then, it calculates the earnings due to each member and distributes the reward accordingly to encourage members’ motivation. The reputation evaluations of team members are uploaded to the blockchain.

### 2.2. Blockchain Architecture

**Figure 2:**
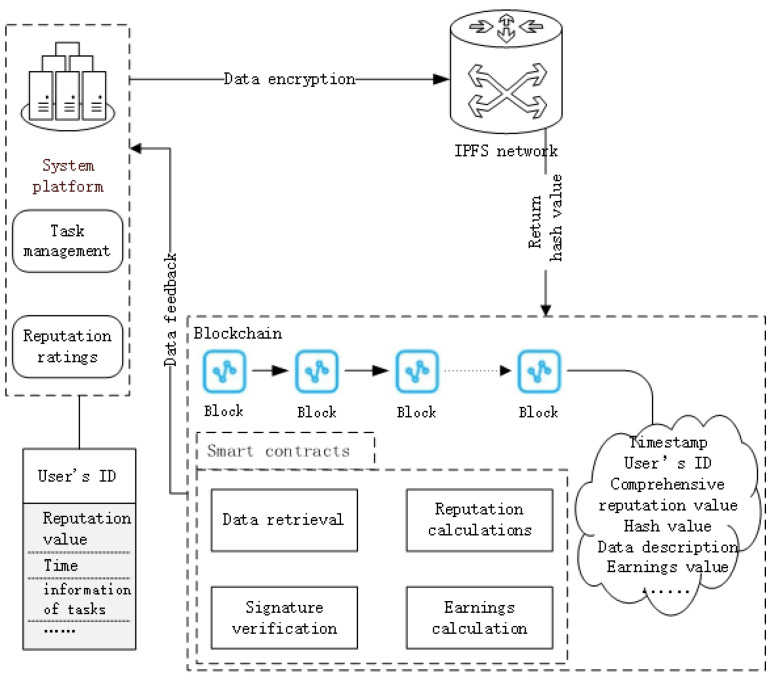
The process of profit distribution and reputation management.

Upon completion of tasks by the team members, the system platform evaluates their reputations based on their task completion status. Then, it encrypts detailed member information, task-related data, and the evaluated reputation values for the current task. These encrypted data are uploaded to the IPFS (InterPlanetary File System) distributed storage system. Moreover, key information about these data, such as data description, data type, and encrypted hash value, is published to the blockchain [15], ensuring the security and reliability of member reputation data. Smart contracts within the blockchain retrieve historical data based on uploaded member IDs. They automatically calculate the comprehensive reputation value using preset rules and algorithms. Subsequently, the smart contracts compute the earnings for each member and write the executed contract result into the block. The earnings data are then sent back to the system platform. As shown in Figure 2, the platform will distribute the income according to the income value of each member, so as to motivate team members to actively participate in the task and maintain a good reputation.

### 2.3. Interaction Diagram

**Figure 3:**
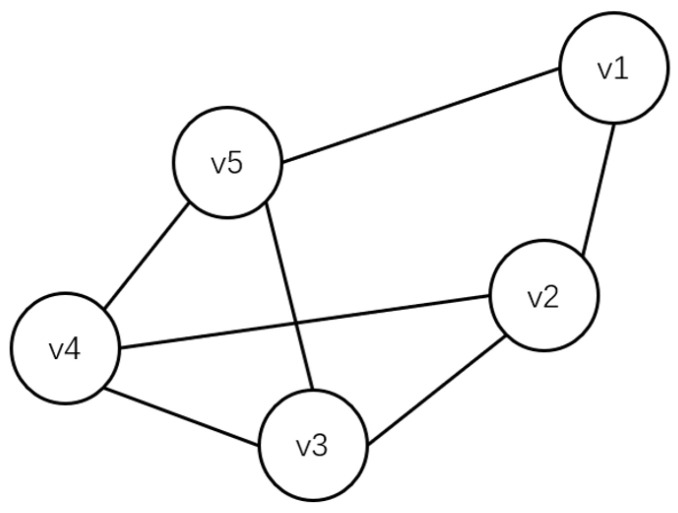
Team Member Interaction Graph.

Based on the collaborative nature among users, the interaction relationships between users can be modeled as an undirected graph [16], denoted as graph G=(V,E), where each user is represented as a node $V=\{v_{1},v_{2},\cdots ,v_{m}\}$ and the set of direct interaction relationships between users is represented as $E=\{\left(v_{1},v_{2}\right),\left(v_{2},v_{3}\right),\cdots ,\left(v_{4},v_{5}\right)\}$. This is illustrated in Figure 3. Through this graphical representation, the relationships and interactions among members of the team can be clearly depicted.

By establishing an undirected graph model of team relationships, a better understanding of the interactions among team members can be gained. This can be applied in computing indirect reputation, comprehensive reputation, and profit allocation. Next, we will further explain how to utilize this undirected graph model to calculate indirect reputation among members and evaluate their contributions within the team. This aims to incentivize members while avoiding free-riding behaviors where certain members benefit from the task outcomes of others.

## 3. Algorithm Design

The focus of this paper is on how to recruit highly collaborative team members within a limited budget, aiming to maximize overall team efficiency. Subsequently, under the given conditions of tasks and team member information, it considers the distribution of the team’s benefits to achieve a fair allocation of earnings among team members, ultimately enhancing their motivation to engage in task execution.

### 3.1. Team Recruitment Mechanism Design

#### 3.1.1. Team Recruitment Mechanism Based on IPA-TRM

Assuming the platform has s users and n tasks, the s users are represented by the set $U=\{u_{1},u_{2},\cdots ,u_{s}\}$, denoted as $u_{k}=\left\{\vartheta_{k}^{i},\zeta_{k}^{i},\delta_{kj},\varkappa_{k}\right\}$, where $\vartheta_{k}^{i}$ represents the perceptual capability of the $u_{k}$ executing task i, indicating the quantity of perceptual data the user can collect for task i. $\zeta_{k}^{i}$ signifies the bidding of $u_{k}$ for task i, while $\varkappa_{k}\subseteq H=\left\{h_{1},h_{2},\ldots ,h_{d}\right\}$ represents the set of task topics that interest the users. The stronger the familiarity between two users, the higher their collaborative capability. Here, $\delta_{kj}$ denotes the collaborative capability between $u_{k}$ and $u_{j}$,$\delta_{kj}\in \left[0,1\right]$ and $\delta_{kj}=\delta_{jk}$.

In the system, the n tasks are represented using the set $T=\{t_{1},t_{2},\cdots ,t_{n}\}$. Each specific attribute of every task can be denoted as $t_{i}=\left\{L_{i},M_{i},B_{i},q_{i},\Psi_{i}\right\}$, where $L_{i}$ represents the task’s posting location, $M_{i}$ indicates the required number of recruited users, $B_{i}$ stands for the task’s budget, and $\Psi_{i}$ represents the set of task topics. $q_{i}$ signifies the data volume threshold set by the task requester, signifying the minimum quantity of perceptual data required. Only when the amount of perceptual data meets this threshold can the publisher analyze and derive certain values from it, such as the distribution of urban air quality, among other aspects. The formal expression of the team recruitment problem is:

(1) $$\max\phi (U_{m},t_{i})$$

(2) $$s.t.\left\{\begin{array}{c} |U_{m}|=M_{i},\\ \sum_{u_{k}\in U_{m}}\zeta_{k}\leq B_{i},\\ Q\left(U_{m},t_{i}\right)\geq q_{i} \end{array}\right.$$

The optimization objective is to maximize the team utility value while satisfying multiple constraints, aiming to allow the requester to spend a lesser budget to gather a greater amount of perceptual data. The first constraint is that the task requester, due to cost, the number of task areas, or other limitations, needs to recruit a specific number of users to complete the perceptual task. The second constraint is that the total cost of collecting perceptual data must not exceed the given task budget. The third constraint is that the quantity of perceptual data collected by the team must not fall below the data volume threshold.

The team’s utility is defined as follows: it represents the quantity of perceptual data that the team can collect per unit cost.

(3) $$\phi \left(U_{m},t_{i}\right)=\frac{1}{M_{i}}\sum_{u_{k}\in U_{m}}\frac{\bar{\delta_{k}}\vartheta_{k}^{i}}{\zeta_{k}^{i}}$$

$\bar{\delta_{k}}=\sum_{w_{j}\subseteq U_{m},j\neq k}\frac{\delta_{kj}}{M_{i}-1}$ is the average collaboration ability of a user $u_{k}$ within the team. $M_{i}$ is the number of users that the task publisher needs to recruit. $U_{m}=\left\{u_{1},u_{2},\cdots ,u_{m}\right\}$ represents a team composed of m users within the platform. The likelihood of a user $u_{k}$ being willing to collaborate with a user $u_{j}$, multiplied by the quantity of perceptual data that $u_{k}$ can collect, estimates the quantity of perceptual data that the user $u_{k}$ can gather when collaborating, expressed in a mathematical formula as $\hat{q_{i}}=\delta_{kj}*\vartheta_{k}$. Expanding this to the team level, the team’s data volume is defined below to estimate the total quantity of perceptual data that the team can gather.

(4) $$Q\left(U_{m},t_{i}\right)=\sum_{u_{k}\subseteq U_{s}}\bar{\delta_{k}}\vartheta_{k}^{i}$$

To maximize the team’s utility, all teams containing $M_{i}$ users are obtained through combinatorial arrangements, aiming to select efficient teams that satisfy the constraints using a global search algorithm. However, due to the time complexity of solving all possible user combinations reaching up to $\left.O\left(\left(\begin{array}{c} \left|S\right|\\ M_{i} \end{array}\right)\right.\times M_{i}\right)$, the global search algorithm cannot efficiently recruit teams for tasks, where |S| represents the number of users in the platform. To enhance the algorithm’s runtime efficiency, the team recruitment problem is transformed into finding the maximum spanning tree problem in graph theory, followed by the utilization of an enhanced Prim’s algorithm to solve it. Firstly, transform the formula for team utility:

(5) $$\begin{array}{cc} \phi \left(U_{m},t_{i}\right) & =\frac{1}{M_{i}}\sum_{u_{k}\in U_{m}}\frac{\bar{\delta_{k}}\vartheta_{k}^{i}}{\zeta_{k}^{i}}\\ & \overset{\delta_{kj}=\delta_{jk}}{⟹}\sum_{u_{k},u_{j}\in U_{m}}\frac{\left(\frac{\vartheta_{k}}{\zeta_{k}}+\frac{\vartheta_{j}}{\zeta_{j}}\right)\delta_{kj}}{M_{i}\left(M_{i}-1\right)} \end{array}$$

Then, the utility contribution brought to the team by any collaboration between two users can be calculated according to the following formula:

(6) $$\pi \left(u_{k},u_{j}\right)=\frac{\left(\frac{\vartheta_{k}}{\zeta_{k}}+\frac{\vartheta_{j}}{\zeta_{j}}\right)\delta_{kj}}{M_{i}\left(M_{i}-1\right)}$$

Construct an undirected weighted graph G=(V,E) based on the utility contributions between users. In graph G, each vertex represents a user, denoted by the vertex set $V=\{v_{1},v_{2},\cdots ,v_{m}\}$; the edge weight between any two vertices $v_{k}$ and $v_{j}$ is recorded as $e_{kj}$; and $e_{kj}=\pi \left(u_{k},u_{j}\right)$. Based on the constructed graph, the team recruitment problem is transformed into the problem of “finding the maximum spanning tree under multi-constraint conditions”, which is formally expressed as follows:

(7) $$\left.\begin{array}{c} \max\sum_{v_{k},v_{j}\in V}e_{kj}\\ s.t.\left\{\begin{array}{cc} & |V^{\prime }|=M_{i},\\ & \sum_{v_{k}\in V}\zeta_{k}\leq B_{i},\\ & Q\left(V^{\prime },t_{i}\right)\geq q_{i} \end{array}\right.. \end{array}\right.$$

**Figure 4:**
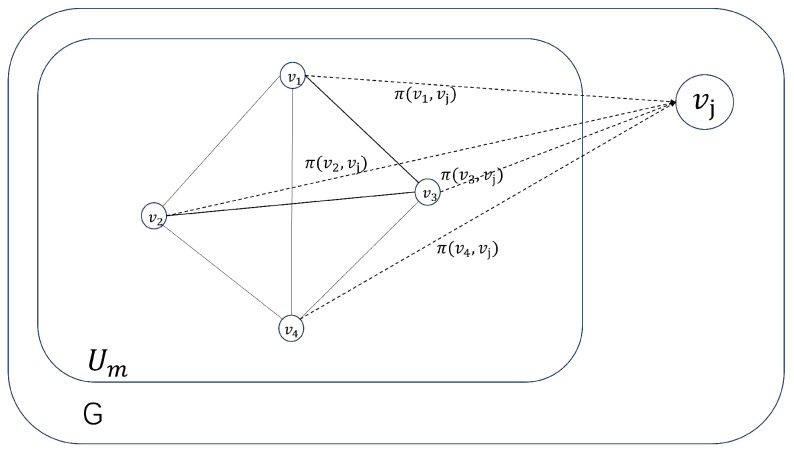
The improved Prim algorithm.

As shown in Figure 4, assuming the team consists of users $v_{1},v_{2},v_{3},$ and $v_{4}$, it is possible to calculate the utility contribution of adding any user $v_{j}$ to $U_{m}$ as $\Delta \left(U_{m},\nu_{j}\right)=\sum_{v_{k}\in U_{m},k\neq j}\pi \left(\nu_{k},\nu_{j}\right)$. The similarity between the set of topics that users are interested in and the set of topics in the task is considered the preference value. However, due to the small preference values obtained through the similarity coefficient calculation, they cannot be directly applied. Hence, a sine function is used to appropriately amplify the results. The calculation formula for the preference value $o_{kj}$ is as follows.

(8) $$o_{ki}=sin\left(\frac{\pi }{2}*\frac{\left|\varkappa_{k}\cap \Psi_{i}\right|}{\left|\varkappa_{k}\right|+\left|\Psi_{i}\left|-\left|\varkappa_{k}\cap \Psi_{i}\right.\right|\right)}\right),o_{ki}\in \left[0,1\right]$$

Based on this, the improved Prim maximum spanning tree algorithm solves the team recruitment problem.

This article makes two modifications to Prim’s maximum spanning tree algorithm: (1) The starting vertex for the maximum spanning tree is selected sequentially instead of the original random selection. As a result, the algorithm will eventually yield |V| Prim’s maximum spanning tree. (2) The termination condition in which the algorithm stops when the number of vertices in the maximum spanning tree reaches $|M_{i}|$.

As shown in Algorithm 1, this paper takes an undirected weighted graph G, the number of recruited users $M_{i}$, the budget $B_{i}$, the data volume threshold $q_{i}$, the task’s topic set $\Psi_{i}$, and the interest preference threshold $o_{0}$ as the algorithm’s inputs, ultimately returning team $U_{m}^{*}$ that satisfies the constraints and possesses the highest utility. Initially, the global variable is initialized, representing the highest team utility found so far (line 1). Users who lack interest in the task are likely to decline even if they possess strong capabilities, so this subset of users is eliminated from the candidates (line 2). Then, iteratively take the vertex $v_{k}$ from the vertex set V as the initial member of team and use the user’s bid $\zeta_{k}^{i}$ as the initial cost teamcost of the team (lines 3–5). Continuously search for users, $v_{j}$, who can contribute the most utility to the team and add them to the team, updating the team’s cost, until the number of team members reaches the upper limit for recruited users, denoted by $|U_{m}|=M_{i}$ (lines 6–10). If the team’s cost exceeds the budget $B_{i}$ or the team’s data volume does not meet the publisher’s specified data volume threshold $q_{i}$, the recruitment for this team is abandoned (lines 11–13). If the utility value of the team $U_{m}$ is higher than the historical record, update the global variable cnt and the best team $U_{m}^{*}$ (lines 14–17). Finally, the team $U_{m}^{*}$ obtained is the solution to Equation (1) (line 19).


**Algorithm 1** Team Recruitment
**Input:** G(V,E); $M_{i}$; $B_{i}$; $q_{i}$; $\Psi_{i}$**Output:** $U_{m}^{*}$;1:cnt=0;2:G ← Calculate the preference value of all nodes in graph G according to Formula (8) and remove nodes with interest preference values smaller than the interest preference threshold;3:
**for all** $v_{k}\in V$ **do**
4:    $U_{m}=$$\left\{v_{k}\right\}$;5:    $teamCost=\zeta_{k}^{i}$;6:    **while** $|U_{m}|<M_{i}$ **do**7:        $v_{j}=\underset{v_{j}\in V\setminus U_{m}}{\arg\max}\sum_{v_{c}\in U_{m}}\pi (v_{j},v_{c})$;8:        $U_{m}=U_{m}⋃\left\{v_{j}\right\}$;9:        $teamCost+=\zeta_{k}^{j}$;10:    **end while**11:    **if** $teamcost>B_{i}\;or\;Q\left(U_{m}\right)<q_{i}$;** then**12:        continue;13:    **end if**14:    **if** $\phi (U_{m},t_{i})>cnt$; **then**15:        $cnt=\phi (U_{m},t_{i})$;16:        $U_{m}^{*}=U_{m}$;17:    **end if**18:
**end for**
19:return $U_{m}^{*}$.



#### 3.1.2. Proof of Algorithm Validity

**Figure 5:**
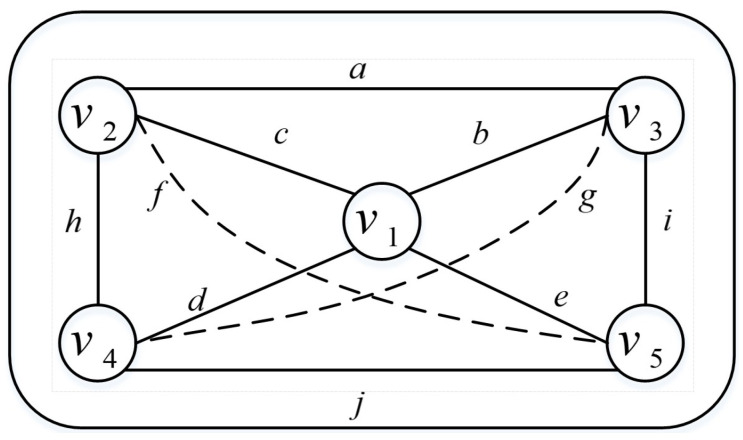
Improved proof of Prim algorithm’s effectiveness.

In this section, we aim to demonstrate that the improved Prim’s maximum spanning tree algorithm can inevitably identify the team with the highest utility. As illustrated in in Figure 5, for convenience in the proof, the letter a∼j represents the collaborative utility contributed by users within the team. The subsequent part will individually prove that recruiting any number of users using the improved Prim’s maximum spanning tree algorithm will always result in the team with the highest utility:

1. When recruiting two users

Assume the team with the highest utility is represented by $U_{m}^{*}=\left\{v_{1},v_{2}\right\}$. Consequently, c>a,c>f,c>h is implied. Considering $v_{2}$ as the root of the maximum spanning tree (as $U_{m}=\left\{v_{2}\right\}$), according to line 7 of Algorithm 1, vertex $v_{1}$ will certainly be added to the set of vertices in the maximum spanning tree, that is, $U_{m}=\left\{v_{2}\right\}$. Thus, it is successfully demonstrated that the team with the highest utility has been identified.

2. When recruiting three users

Suppose team $U_{m}^{*}=\left\{v_{1},v_{2},v_{3}\right\}$ has the highest utility, and a⩾b⩾c. In the worst-case scenario, when considering $v_{2}$ and $v_{3}$ as roots of the maximum spanning tree, $U_{m}^{*}$ is not found. Thus, it suffices to prove that choosing $v_{1}$ as the root of the maximum spanning tree will definitely find $U_{m}^{*}$:

(1) Assuming $v_{2}$ as the root of the maximum spanning tree, and the next vertex, any one of $U_{m}^{*}$, to be added is $v_{4}$, then h>a. Let $h=a+\varepsilon_{1}$, $\varepsilon_{1}>0$. Since $U_{m}^{*}$ has the highest utility value, a+b+c>h+c+d, implying a+b>h+d. Also, considering $h=a+\varepsilon_{1}$, a+c>d+g. Similarly, a+c>d+g.
(2) Assuming $v_{3}$ as the root of the maximum spanning tree, and the next vertex, any one of $U_{m}^{*}$, to be added is $v_{5}$, then i>a. Let $i=a+\varepsilon_{2}$, $\varepsilon_{2}>0$. Since $U_{m}^{*}$ has the highest utility value, a+b+c>b+e+i, implying a+c>e+i. Also, considering $i=a+\varepsilon_{2}$, $e<c-\varepsilon_{2}$.
(3) Next, take $v_{1}$ as the root of the maximum spanning tree, that is, $U_{m}=\left\{v_{1}\right\}$. Because $\left(\begin{array}{c} U_{m},v_{3} \end{array}\right)=b>\sigma \left(\begin{array}{c} U_{m},v_{2} \end{array}\right)>\sigma \left(\begin{array}{c} U_{m},v_{5} \end{array}\right)=e$ and $\sigma (U_{m},\nu_{3})=b>d$, the next vertex added to the maximum spanning tree must be $v_{3}$, which implies $U_{m}=\left\{v_{1},v_{3}\right\}$. Also, because $\sigma \left(U_{m},\nu_{2}\right)-\sigma \left(U_{m},\nu_{5}\right)=a-i+c-e>a-i+\varepsilon_{2}=2\varepsilon_{2}>0$ and $\sigma \left(U_{m},\nu_{2}\right)-\sigma \left(U_{m},\nu_{4}\right)=\left(a+c\right)-\left(d+g\right)>0$, the next vertex added to the maximum spanning tree must be $v_{2}$. Therefore, $U_{m}=\left\{v_{1},v_{3},v_{2}\right\}=U_{m}^{*}$, and we have successfully found the team with the highest utility.

3. When recruiting more than three users

Once we determine the root node $v_{i}$ of the maximum spanning tree, the algorithm will select the next node $v_{j}$ to be added to the maximum spanning tree. We combine $v_{i}$ and $v_{j}$ into a composite node $v_{k}^{*}=\left\{v_{i},v_{j}\right\}$ and calculate the utility contribution of other vertices to the composite vertices by $\gamma \left(\nu_{k}^{*},\nu_{x}\right)=\sum_{\nu_{t}\in \nu_{k}^{*}}\gamma \left(\nu_{t},\nu_{x}\right)$. Assuming $U_{m}=\left\{\begin{array}{c} v_{i},v_{j} \end{array}\right\}$, $U_{m}^{*}=\left\{\nu_{k}^{*}\right\}$, for any vertex $\nu_{x}\in V$,$\gamma \left(U_{m}^{*},v_{x}\right)=\gamma \left(v_{i},v_{x}\right)+\gamma \left(v_{j},v_{x}\right)=\gamma \left(U_{m},v_{x}\right)$, so $U_{m}^{*}$ and $U_{m}$ are equivalent, and the vertex combination will not affect the selection of subsequent vertices. Therefore, when recruiting more than three users, we can transform the proof problem into a case in which the user quantity is equal to three by merging the vertices. As the proof for the case of three users has been provided earlier, we can conclude that when recruiting more than three users, $U_{m}^{*}$ will also be found, which represents the team with the highest utility. In conclusion, the optimality is proved.

### 3.2. Research on Team Incentive Mechanism

In traditional crowdsensing, tasks are usually completed independently by individuals, and the completion of tasks is influenced by individual-related factors. Correspondingly, the incentive mechanism typically evaluates the completion of user tasks, forming the user’s direct reputation value based on this assessment. In collaborative crowdsensing, tasks are accomplished through mutual cooperation among users, and task completion is influenced not only by individual factors but also by the constraints of collaborative partners. Consequently, the incentive mechanism needs to consider not only the direct reputation value of users but also the indirect reputation value that measures the interaction and collaboration within the team.

Furthermore, the computation of direct reputation requires evaluating the quality of user tasks, which is limited by resources (such as limited computing resources, network bandwidth constraints, time sensitivity, etc.). Therefore, the platform cannot perform real-time detection of the quality of participants’ task completions and must conduct a quality estimation of the task data submitted by users in advance.

#### 3.2.1. Task Quality Assessment

Assuming there are L levels of data quality, L={1,2,…,L}, each corresponding to a specific variance $\sigma_{l}^{2}$ and data quality $q_{l}$. *l* varies, leading to different variances accordingly. When the user-submitted data correspond to the data quality level $l\left(\begin{array}{c} l\in L \end{array}\right)$, the collected data by the user follows a Gaussian distribution $N(\mu_{j},\sigma_{l}^{2})$ with a data quality of $q_{l}\in \left(0,1\right)$. In a Gaussian distribution, when the mean is the same, a larger variance leads to a flatter distribution, implying greater data dispersion and a higher likelihood of deviation from the true value. Variance is used to measure the concentration of data; different quality levels correspond to Gaussian distributions with different variances. To put it simply, the data quality level determines the concentration of the Gaussian distribution’s dataset. Higher data quality corresponds to a smaller variance, indicating stronger data concentration, thereby making the data closer to the true value.

To more accurately assess the quality of a user’s data, multiple sets of data collected by the user for the same task are used. Specifically, for each dataset, its corresponding data quality is calculated, and these values are summed up and averaged to derive a more precise data quality for the user. As the volume of data collected by the user increases, the system’s estimation of the user’s data quality becomes more accurate. Next, we will detail how to estimate data quality using a single set of perceptual data.

Firstly, as outlined in reference [17] with Algorithm 1, it is necessary to compute the true values of data quality for each task. Subsequently, based on these true values, the distribution of user data quality levels $\left\{\begin{array}{c} \Pi (l),l\in L \end{array}\right\}$ is further determined. In this distribution, Π(l) represents the probability that the user belongs to a data quality level *l*, satisfying the condition $\sum_{l=1}^{L}\Pi \left(l\right)=1$. With this data quality level distribution, it becomes feasible to determine the data quality level corresponding to each set of data. Finally, based on the data quality level of each set of perceptual data, an evaluation of the task’s data quality can be made.

For each task, there exists a ground truth value μ, but there is a certain deviation between the data collected by users and this ground truth value, thereby introducing a bias value $y_{ij}=x_{ij}-\mu_{j}$. User *i* collects data $x_{i^{*}}=\left\{x_{i1},x_{i2},\ldots ,x_{in}\right\}$ corresponding to a deviation value $y_{i^{*}}=\left\{y_{i1},y_{i2},\ldots ,y_{in}\right\}$. The deviation value y for a given task comes from a mixture of L data quality levels, derived from a Gaussian mixture model (GMM). Its probability density function is as follows:

(9) $$p_{\theta }\left(y\right)=\sum_{l=1}^{L}\Pi \left(l\right)N\left(y;0,\sigma_{l}^{2}\right),1\leq l\leq L$$

$\left.\theta =\left\{\Pi \left(\begin{array}{c} l \end{array}\right)\right.,l\in L\right\}$ is an unknown parameter, utilizing a 1×L dimensional random variable $Z_{j}=\left\{z_{j1},z_{j2},\ldots ,z_{jL}\right\}$ to denote the potential data quality level where the deviation value $y_{j}$ may reside, $\sum_{l=1}^{L}z_{jl}=1$, $z_{jl}\in \left\{0,1\right\}$. For such a sample set (Y, Z), the corresponding likelihood function is:

(10) $$\begin{array}{cc} \; & L\left(\theta \right)=\prod_{j=1}^{N}p\left(y_{j},Z_{j}\right)\\ \; & =\prod_{l=1}^{L}\prod_{j=1}^{N}\Pi {\left(l\right)}^{z_{jl}}N{\left(y_{j};0,\sigma_{l}^{2}\right)}^{z_{jl}} \end{array}$$

Use the logarithm:

(11) $$\mathrm{lnL}\left(\theta \right)=\sum_{l=1}^{L}\sum_{j=1}^{N}z_{jl}\left[\ln\pi \left(l\right)+\mathrm{lnN}\left(y_{j};0,\sigma_{i}^{2}\right)\right]$$

Due to the unknown Z, which cannot be directly solved for the parameter θ, the EM algorithm is used here for iterative computation.

(12) $$\begin{array}{cc} Q\left(\theta |\theta^{\left(k\right)}\right)=\sum_{l=1}^{L}\sum_{j=1}^{N}E\left(z_{jl}\right) & \left[\ln\Pi \left(l\right)+\ln N\left(y_{j};0,\sigma_{l}^{2}\right)\right] \end{array}$$

The EM algorithm Expectation (E) step is as follows:

(13) $$E\left(z_{jl}\right)=\frac{\Pi {\left(l\right)}^{\left(k\right)}N\left(y_{j};0,\sigma_{l}^{2}\right)}{\sum_{l=1}^{L}\Pi {\left(l\right)}^{\left(k\right)}N\left(y_{j};0,\sigma_{l}^{2}\right)}$$

The Maximization (M) step of the EM algorithm involves introducing Lagrange multipliers under the constraint $\sum_{l=1}^{L}\Pi \left(l\right)=1$, constructing the Lagrangian function, and solving for parameters $\theta^{\left(k\right)}$ that maximize the given value $Q(\theta |\theta^{\left(k\right)})$.

(14) $$L\left(\theta |\lambda \right)=Q\left(\theta |\theta^{\left(k\right)}\right)+\lambda \left(\sum_{l=1}^{L}\Pi \left(l\right)-1\right)$$

The final step involves taking partial derivatives with respect to each Π(l), l∈L, setting the derivatives to zero, and cumulatively solving the equations for L instances.

(15) $$\Pi {\left(l\right)}^{\left(k+1\right)}=\frac{1}{N}\sum_{j=1}^{N}E\left(z_{jl}\right)$$

The iterative process involves executing the aforementioned E step and M step until convergence is achieved, obtaining the data quality level distribution $\theta^{\left(k+1\right)}=\left\{\Pi {\left(l\right)}^{\left(k+1\right)},l\in L\right\}$ for each user. In conclusion, the data quality level achieved by the users for the tasks is:

(16) $$L_{j}=argmax\left(\Pi \left(l\right)*N\left(y_{j};0,\sigma_{l}^{2}\right)\right),\forall l\in L$$

#### 3.2.2. Reputation Calculations

(1) Direct reputation

To more accurately gauge a user’s individual performance in a task, the direct reputation value of the user is formed by combining the reputation evaluations from historical tasks and the current task.

Based on the completion quality $L_{k}$ of the member $u_{k}$ in the task, the platform evaluates their reputation using the following formula:

(17) $$r_{k}^{now}=\log_{2}\left(L_{k}+1\right)$$

This article normalizes reputation values within the range of [0, 1], where 0 represents complete lack of trustworthiness and 1 signifies absolute trustworthiness. Based on the user’s reputation value derived from the data quality, historical task reputation values are obtained. The Gompertz function is employed to evaluate the user’s historical reputation value $r_{k}^{his}$, as per the following formula:

(18) $$r_{k}^{his}=G\left(G^{-1}\left(r_{k}^{j}\right)+r_{k}^{now}\right)$$

where $G\left(x\right)=e^{-e-\lambda x}$, λ represents the growth rate parameters controlling the speed of reputation convergence, and $r_{k}^{j}$ represents the reputation evaluation of the task number *j*. Assuming $r_{k}^{his}\in \left[0,0.5\right)$, if the member $u_{k}$ consistently enhances the data quality, their historical reputation value can increase rapidly, and their rate of increase will also accelerate. Conversely, if $r_{k}^{his}\in \left[0.5,1\right]$, due to limited room for improvement in historical reputation values, the rate of increase will gradually slow down until it stabilizes.

By integrating historical task reputations of the member $u_{k}$, the weighted sum of historical reputation values for each task in $hisTask=\{t_{1},t_{2},\cdots ,t_{h}\}$ is calculated as follows:

(19) $$\tau_{k}=\sum_{j=1}^{h}\Omega^{\left(n-j+1\right)}r_{k}^{his}$$

$\tau_{k}$ represents the weighted sum of member k’s historical reputation values. Ω is a constant within the range (0, 1] used to differentiate the importance of various historical reputation values. The closer the completion time of the perception task, the higher the weight of its historical reputation value. Based on the weight of member k’s historical reputation values, the direct reputation value is calculated as follows:

(20) $$DR_{k}=\frac{1}{1+e^{-\tau_{k}}}$$

(2) Indirect reputation

As there are no direct evaluations among team members and there exists variability among members, different weights should be allocated when each member provides indirect reputation evaluations to the target user based on their own influence. The weight of indirect influence depends on the member’s direct reputation, the importance of each member in the interaction graph, and the similarity between members.

a. Importance

When calculating the weight of indirect reputation, it is necessary to distinguish between the importance of members within the team and calculate their indirect influence on other members’ reputations. Therefore, based on the network topology, the influence of the members of the team is categorized into two types: local influence and global influence. Local influence reflects a member’s ability to influence their adjacent members, while global influence reflects a member’s influence on all members within the entire team.

When quantifying the local influence of the member $u_{k}$, two factors are considered: the influence of $u_{k}$ on its adjacent members and the clustering coefficient. The influence of the member $u_{k}$ on the member $u_{j}$ is determined by the degree of $u_{j}$, denoted as 1/D(j). The clustering coefficient [18] $C\left(u_{k}\right)=\frac{Ne\left(u_{k}\right)}{D\left(k\right)\left(D\left(k\right)-1\right)}$ of the member $u_{k}$ reflects the tightness of connections between its adjacent members, where $Ne\left(u_{k}\right)$ represents the number of edges among $u_{k}$’s adjacent members in the interaction graph. A larger clustering coefficient indicates more interactions among $u_{k}$’s adjacent members. Therefore, the formula to quantify the local influence of a member is as follows:

(21) $$local\left(u_{k}\right)=\left(1-g\right)C\left(u_{k}\right)+g\sum_{u_{j}\in N\left(u_{k}\right)}\frac{1}{D\left(j\right)}$$

where g∈(0,1) is a predefined weight coefficient, D(j) represents the degree of member $u_{k}$, and $N(u_{k})$ is the set of adjacent members of member $u_{k}$.

The global influence of a member is the member’s ability to influence all other members of the entire team, which is essentially quantifying its importance in the team’s interaction graph. The PageRank algorithm [19] measures the importance of web pages in a network. In this algorithm, the PageRank value of a page signifies its significance; a higher value indicates greater importance. Therefore, using the PageRank value equivalently represents the global influence of each member node. The formula is as follows:

(22) $$PR_{k}=\varepsilon *\Sigma \frac{PR_{j}}{D\left(j\right)}+\frac{1-\varepsilon }{M}$$

where ε is a damping factor between 0 and 1 used to balance the weight between random interaction and interaction with neighboring members. $PR_{k}$ represents the PageRank value of the member $u_{k}$. $PR_{j}$ represents the PageRank value of the other member $u_{j}$ connected to the member $u_{k}$. *M* represents the total number of team members.

The formula for quantifying global influence is as follows:

(23) $$global\left(u_{k}\right)=PR_{k}$$

The comprehensive formula for quantifying a member’s importance in the team, taking into account both their local influence and global influence, is as follows:

(24) $$\begin{array}{ccc} & PR_{k}^{\prime }=\varpi^{*}\left(\left(1-g\right)C(u_{k})+g\sum_{u_{j}\in N\left(u_{k}\right)}\frac{1}{D\left(j\right)}\right) & +\left(1-\varpi \right)global(u_{k}) \end{array}$$

where ϖ∈(0,1) is a predefined weight coefficient.

Finally, the member’s level of importance is transformed into their indirect reputation impact weight on other members using the Sigmoid function, mapped to the target range (0,R], where *R* represents the level of importance.

(25) $$P_{k}=\frac{R}{1+e^{\left(-\varsigma^{*}PR_{k}^{\prime }-\left(R/2\right)\right)}}$$

$P_{k}$ can be used to control the weight of calculating the indirect reputation among users, while ς is a parameter controlling the shape of the curve.

b. Similarity calculation based on Jaccard coefficients

To provide a more comprehensive and accurate assessment of the influence of member relationships on indirect reputation, a member similarity indicator has been introduced to further compute the weight contribution of members to each other’s indirect reputation. If the target member and the team members exhibit a high level of similarity, the indirect reputation generated by that team member is more reliable, thus contributing more to the reputation of the target member. The Jaccard coefficient is utilized to measure the similarity among team members who have participated in the same team tasks in previous assignments, evaluating a team member’s reputation contribution to others. The formula for this calculation is as follows:

(26) $$S_{i,k}=\frac{\left|Tas(i)\cap Tas(k)\right|}{\left|Tas(i)\cup Tas(k)\right|}$$

where $S_{i,k}$ represents the similarity between the member $u_{i}$ and the member $u_{k}$, Tas(i) indicates the set of tasks completed by the member $u_{i}$ in the past, and Tas(k) denotes the set of tasks completed by the member $u_{k}$ in the past.

c. Indirect reputation weighting

Define a variable χ∈{0,1} to indicate whether there is interaction between two members. When there is no interaction between two members, it is denoted by χ=0; otherwise, it is denoted by χ=1. Using the above PageRank and similarity calculation formulas, compute the reputation contribution weight of each member to $u_{k}$ in order to calculate the indirect reputation of the member $u_{k}$. The weight calculation formula is as follows:

(27) $$\omega_{i}=\frac{DR_{i}P_{i}S_{i,k}\chi_{i,k}}{\sum_{m-1}DR_{i}P_{i}S_{i,k}}$$

where $\omega_{i}$ represents the reputation contribution weight of the member $u_{i}$ to the target member $u_{k}$, $DR_{i}$ denotes the direct reputation of the member $u_{i}$, $P_{i}$ represents the importance level of the member $u_{i}$ in the team, $S_{i,k}$ indicates the similarity between the target member $u_{k}$ and member $u_{i}$, and m−1 signifies the set of all members except the target member $u_{k}$.

Using the aforementioned calculations, the indirect reputation of the member $u_{k}$ can be derived as follows:

(28) $$IR_{k}=\sum_{m-1}\omega_{i}DR_{i}$$

By taking into account the interactive dynamics, reputation contributions, and similarity between team members, a more accurate assessment of the indirect contribution level of each member within the team has been achieved.

(3) Comprehensive reputation

The more team members interact with a member, the higher the weight of their indirect reputation. Therefore, the weight β of the direct reputation should decrease as the number of interacting members increases and have a lower bound. The improved arctan function is used to compute the weight of the direct reputation:

(29) $$\beta =\frac{2\left(1-\beta_{1}\right)}{\pi }arc\cot\left(\frac{N^{\prime }}{\pi \beta_{2}}\right)+\beta_{1}$$

The parameters are defined as follows: $\beta_{1}$ controls the lower bound of the weight of direct reputation values, $\beta_{2}$ controls the rate at which the weight of direct reputation decreases as the number of interactions increases, $\beta_{2}$ is the input number of interacting members, and β outputs the weight of the direct reputation value.

The comprehensive reputation is calculated by considering both the direct and indirect reputations of members in the team task, aiming to assess their contributions and allocate appropriate rewards. The formula for calculating the comprehensive reputation is as follows:

(30) $$Re_{k}=\beta DR_{k}+\left(1-\beta \right)IR_{k}$$

The variables in the formula represent the following: $Re_{k}$ stands for the comprehensive reputation of the member $u_{k}$, $DR_{k}$ denotes the direct reputation of the member $u_{k}$, $IR_{k}$ represents the indirect reputation of the member $u_{k}$, and β signifies the weight of the direct reputation value.

#### 3.2.3. Member Earnings Incentives

The interaction undirected graph of team members $U_{m}=\left\{u_{1},u_{2},\cdots ,u_{m}\right\}$ is transformed into a weighted undirected graph, considering the collaborative abilities among members. For any two nodes $u_{k}$ and $u_{j}$ in the graph, the edge weight is computed based on their comprehensive reputation values and collaborative abilities.

(31) $$w_{kj}=\frac{\left(Re_{k}+Re_{j}\right)\delta_{kj}}{m-1},\forall u_{k},u_{j}\epsilon U_{m},k\neq j$$

The characteristic function $V\left(k\right)=\frac{w_{kj}}{2}$ represents the contribution made by each member in this collaborative project.

The Shapley value is a method for fairly distributing resources based on cooperative game theory concepts. In this approach, the Shapley values consider the contributions of each member by calculating the marginal contribution of each member to allocate the final alliance value, ensuring fair rewards for their contributions. In a team with a total of m members, the Shapley value for each member is denoted as $\varphi_{k}$, and the total team payoff is V(M). According to the concept of Shapley values [20], as they represent each member’s contribution to the team, the total payoff can be distributed based on the Shapley values of each member, thereby discouraging free-riding behavior within the team.

The formula for the Shapley values is as follows:

(32) $$\varphi_{k}=\sum_{S\subset N\setminus \{k\}}\frac{\gamma {!}^{*}\left(m-\gamma -1\right)!}{m!}*\left(V\left(S\cup \{k\}\right)-V\left(S\right)\right)$$

where *m* is the total number of team members, *S* is the subset of members excluding member *k*, and γ is the size of the subset *S*, that is, the number of participants included in *S*. V(S∪k) is the alliance income after adding participant *k* to the subset *S* to form a new subset.

Therefore, the derived earnings that each member should receive can be expressed as follows:

(33) $$R_{k}=\frac{\xi_{k}+\varphi_{k}\frac{\Sigma \xi_{k}}{\Sigma \varphi_{k}}}{\Sigma \left(\xi_{k}+\varphi_{k}\frac{\Sigma \xi_{k}}{\Sigma \varphi_{k}}\right)}*V\left(M\right)$$

where $\xi_{k}$ and $\varphi_{k}$ represent the bid and Shapley value of each member, respectively. By taking the weighted average of bids and Shapley values for each member and dividing by the sum of the weighted averages of all members, we obtain the overall earnings distribution ratio for each member. Then, the total earnings V(M) are allocated to the members based on this ratio.

However, members’ negative behaviors disrupt the collaborative efficiency of the team task execution, hindering the completion of team tasks. In this section, a penalty factor is introduced for members’ negative factors. Assuming that in the team task, the overall loss of earnings due to subjective negative behaviors, such as passive cooperation by a member, is represented as LOSS, and the loss caused by member *k* is represented as loss, the penalty factor for negativity can be calculated as:

(34) $${℧}_{k}=loss/LOSS$$

Then, the earnings obtained by member $u_{k}$ at this point would be:

(35) $$R_{k}^{\prime }=R_{k}+\left[\frac{1/{℧}_{k}}{\sum_{k=1}^{m}\left(1/{℧}_{k}\right)}-\frac{1}{m}\right]R_{k}$$

By multiplying the negative penalty factor by the share of each member in the profit distribution, the impact of negative behavior on earnings can be reduced, thus allowing for a fairer distribution of profits among the team members involved in the task.

## 4. Experiment

### 4.1. Experimental Data

1. Dataset

The dataset U.Rovira i Virgili used in the experiment represents the email communication network of the Rovira i Virgili University in Tarragona, southern Catalonia, Spain. This dataset consists of 1133 nodes representing users and 5451 edges, indicating at least one email communication between the connected users. It does not store the direction or quantity of emails exchanged between individuals.

2. Basic settings

This experiment considers the top 250 users ranked by node degree as the users on the crowdsensing platform. Using the edge data from the dataset, the interactions among these 250 users can be obtained. The dataset is transformed into experimental data through the following procedure:

(1) The perceptual ability (ϑ) of users is replaced with their degrees in the interactions.
(2) The collaboration matrix, represented as δ, is initialized based on the interactions among the 250 users. If there is interaction between users $u_{k}$ and $u_{j}$, they will have a higher collaboration ability, setting $\delta_{kj}$ as a random value between [0.5, 1]; otherwise, it will be a random value between (0, 0.5).
(3) The total number (M) of users on the platform: M∈[50,250].
(4) The number (N) of recruited team members: N∈[1,M].

Simulate user bids for the task through Gaussian sampling with an expected value of 10 and a standard deviation of 3. This is performed to replicate the scenario where the majority of users provide honest bids while a minority might overstate their costs in anticipation of higher rewards. Additionally, each user’s preference is set to follow a uniform distribution over [0, 1]. Based on the statistical data from 250 users, the average perceptual ability is approximately 7. To facilitate the experiment, the default data volume threshold for tasks ($q_{i}$ ) is set to 7∗N.

3. Evaluate metrics

In the collaborative crowdsensing system, this paper introduces two evaluation metrics for assessing experimental effectiveness:

(1) Team Utility Value Metric: This refers to the ratio of the actual amount of perceptual data collected by the team to the total cost incurred by the team, representing the quantity of perceptual data acquired per unit cost.
(2) Fairness: Refers to the fairness and equity in income distribution, ensuring each member receives fair and proportional remuneration according to their contributions. Jain’s fairness index is used to measure fairness, and its calculation method is as follows:

$$F=\frac{{\left(\sum_{i=1}^{n}x_{i}\right)}^{2}}{n\Sigma_{i=1}^{n}x_{i}^{2}}\in \left[\frac{1}{n},1\right]$$

The greater the value of F, the higher the level of fairness.

### 4.2. Experimental Results and Analysis

1. Comparison of the results of team recruitment

**Figure 6:**
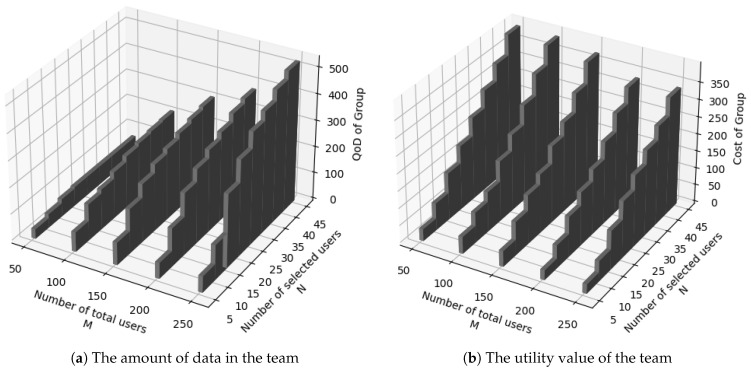
Analysis of the effectiveness of the team recruitment algorithm.

The paper sets the task budget at 500, the total number of users on the platform (M) varies from 50 to 250, and the number of recruits (N) varies from 5 to 45. The experimental results are shown in Figure 6. Figure 6a shows the variation in the team’s data volume. An increase in M implies an expanded range of users that the algorithm can select, while an increase in N means a larger number of users from which the algorithm can choose. Therefore, it is reasonable that the recruited data volume of the team shows an upward trend. Figure 6b demonstrates the changes in the team’s utility. With the growth of M, the overall utility of the team shows an upward trend. This is due to the team recruitment algorithm proposed in this paper consistently discovering and recruiting teams with higher utility.

**Figure 7:**
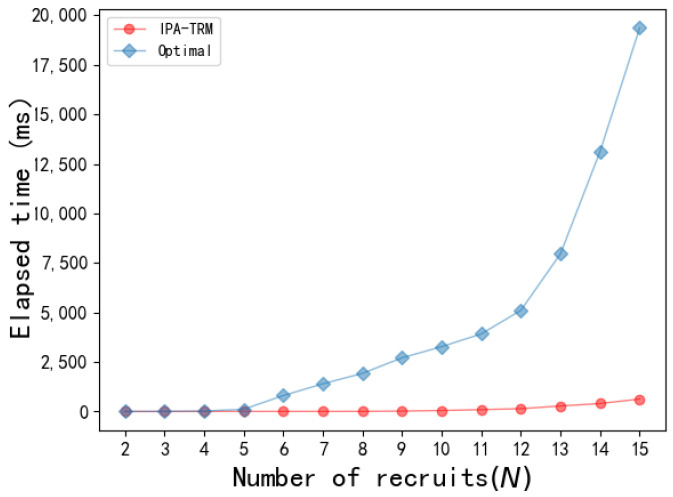
Comparison of IPA-TRM and Optimal runtimes.

The algorithm’s runtime is a critical performance metric for measuring an algorithm’s efficiency. This paper compares the time costs between the IPA-TRM algorithm and the global search algorithm Optimal. Initially, with a task budget set at 500 and a total platform user count of 30, the recruited user count was increased from 2 to 15. The experimental results, as shown in Figure 7, show that when N increases from 2 to 5, there is little difference in the time cost between the two algorithms. However, as N increases from 5 to 12, the time cost gap between the Optimal and IPA-TRM algorithms gradually widens. When N>12, Optimal’s time cost experiences a sharp increase, while the IPA-TRM maintains only a minimal increase. Therefore, the IPA-TRM outperforms Optimal in algorithm performance.

2. Team member motivation

The main parameters of this experiment are given in Table 1.

(1) Dynamic reputation assessment

**Figure 8:**
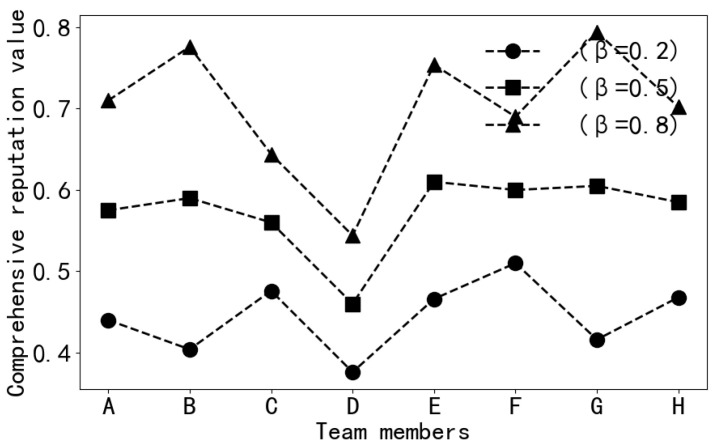
The impact of different β parameters on comprehensive reputation.

In this experiment, the parameter β is set to values of 0.2, 0.5, and 0.8. As shown in Figure 8, each curve represents the impact of different β values on the members’ comprehensive reputation:

(a) β = 0.2: In this scenario, comprehensive reputation is primarily determined by indirect reputation. With a decrease in the β value, the comprehensive reputation is relatively lower. This suggests that when considering comprehensive reputation, it heavily relies on the influence of team members throughout the team interaction, while the weight of direct reputation is lower.
(b) β = 0.5: In this case, the weights of direct and indirect reputations are equal. Comprehensive reputation balances direct and indirect reputations, leading to a smoother change in the comprehensive reputation values in the graph.
(c) β = 0.8: Here, comprehensive reputation is mainly determined by direct reputation. As seen, with an increase in the β value, the comprehensive reputation is relatively higher. This means that when considering comprehensive reputation, it relies more on direct positive feedback received directly from the platform, while the weight of indirect reputation is lower.

In conclusion, by adjusting the β value, it is possible to balance the weight of direct and indirect reputations in the comprehensive reputation according to the actual situation.

(2) Member Earnings Analysis

**Figure 9:**
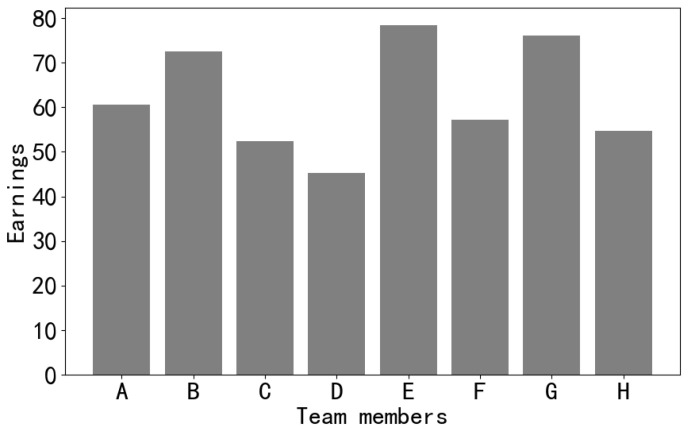
Chart of member earnings.

In this experiment, the total profit for the team was set at 500, and the total loss incurred by the team was 50, as shown in Figure 9. Through the analysis of members’ earnings:

- Discrepancy in earnings: The bar chart demonstrates variations in the earnings of different members, indicating potential differences in contributions among them. There appears to be a positive correlation among members’ earnings.
- Relationship between contribution and earnings: There is a positive correlation between the contributions made by members and their respective earnings.
- Importance and earnings: Observing the charts, it becomes evident that the member with the highest earnings tends to be the one contributing the most. Hence, differentiating between members based on their contributions further emphasizes the significance of comprehensive reputation values in income distribution.

(3) Comparative Analysis of Member Earnings and Average Returns

**Figure 10:**
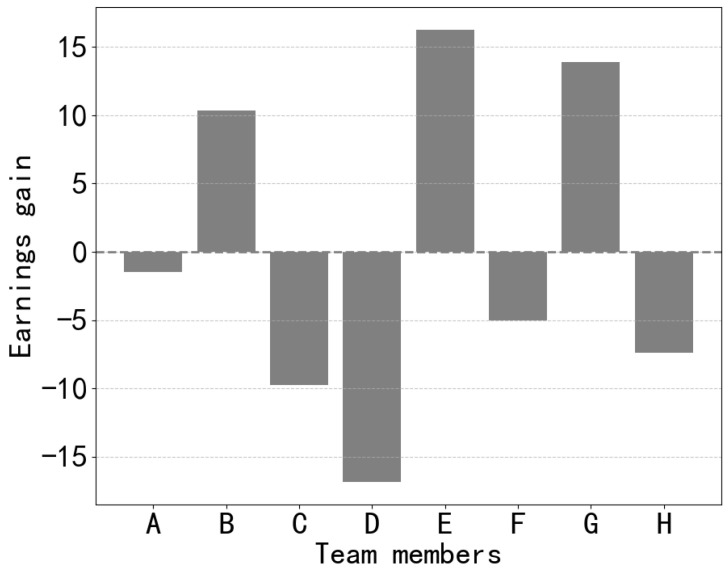
Comparison of member earnings with the average.

The gain in earnings refers to the difference between each member’s earnings and the average earnings, calculated as earnings minus the mean earnings. By computing the gain in earnings, a clearer view emerges regarding the extent to which each member’s earnings deviate from the team’s average earnings. As shown in Figure 10, members B, E, and G exhibit positive gains in earnings, indicating that they have earned higher than the average level. Members A and F show gains in earnings close to the average level, suggesting their contributions align closely with the team average, leading to a relatively equitable distribution of earnings. On the contrary, members C, D, and H display negative gains in earnings, implying that they have earned less than the average level. This might suggest lower contributions from them or, to some extent, a negative impact on the team’s performance.

Therefore, for both negative and positive contributors, their gains in earnings exhibit two extremes. Positive contributors make higher contributions, resulting in a positive growth in earnings. Conversely, negative contributors experience negative growth in earnings. Members with positive gains tend to maintain their positivity, whereas those with negative gains might become more proactive in completing tasks to earn more rewards. This could act as a form of motivation to some extent.

(4) Fairness analysis

This paper utilizes Jain’s fairness index to evaluate whether the MR-SVIM adheres to the principle of fairness. It compares various allocation methods to validate their feasibility and superiority.

This study selects 8 teams as subjects for analysis and compares the proposed incentive method, the MR-SVIM, with the following algorithms to assess their fairness in profit distribution:

(1) Equal Distribution Method: A traditional approach that evenly distributes a certain quantity or resource among a specific number of individuals or entities.
(2) VCG Mechanism [21]: An effective resource allocation method among multiple participants that encourages participants to honestly report their true preferences and information.
(3) MTRPM Method: The method, known as the Myersonian Truthful Reporting Payment Mechanism (MTRPM), is based on the Myerson theory. It involves initially ranking the users within the team based on their utility contribution values, removing the user with the lowest utility value, and subsequently selecting another user from the remaining pool who can provide the maximum utility contribution to the team. The payment formula is as follows:

$$p_{k}^{i}=\left\{\begin{array}{c} \frac{\bar{\delta_{k}}\vartheta_{k}}{\bar{\delta_{K^{*}}}\vartheta_{K^{*}}}\zeta_{K^{*}},k\in U_{m}\\ 0,k\notin U_{m} \end{array}\right.$$

The parameter $p_{k}^{i}$ is known as the critical payment value. When the user $u_{k}$’s bid is less than or equal to the critical payment value, they are eligible to be selected, and the payment is made according to this critical payment value. However, if the user $u_{k}$’s bid exceeds the critical payment value, the platform will select $u_{k^{*}}$, who offers a higher utility contribution, to perform the task, resulting in a payment of zero for the user $u_{k}$.

**Figure 11:**
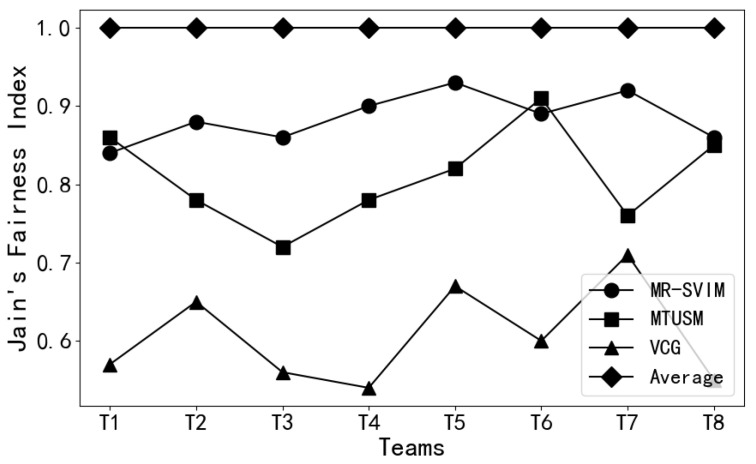
Comparison of the fairness of income distribution under the four methods.

According to the definition of Jain’s fairness index, a higher index indicates greater fairness. As shown in Figure 11, both the MTRPM and MR-SVIM methods exhibit better fairnesses compared to the VCG mechanism, and the VCG mechanism shows unstable performance and poor fairness. However, in comparison to the MTRPM method, the MR-SVIM method demonstrates more stability in fairness. The significant fluctuations observed in the MTRPM method are mainly due to its consideration solely of members’ bids and collaborative perception abilities without taking into account their actual contributions within the team. This limitation results in substantial disparities in the Jain’s fairness index. Conversely, the MR-SVIM method combines members’ contributions and bids in distributing the overall team utility, ensuring that members’ earnings are proportional to their contributions. Consequently, it exhibits better performance in fairness indicators and shows less variability.

Moreover, although the Average Distribution method attains the highest fairness score, it neglects the actual contributions of individual members, treating all contributions as equal. Therefore, this method is only suitable for straightforward scenarios.

In summary, considering the motivation of incentivizing members, the MR-SVIM method, which integrates members’ contributions and other factors, demonstrates favorable characteristics in terms of fairness and stability. It takes into account the actual contributions of members within the team, thereby promoting a fairer and relatively stable distribution of earnings.

## 5. Conclusions

In this paper, we not only consider recruiting users based on individual capabilities but also place significant emphasis on recruiting users with strong collaboration skills and contributions to the overall team utility. Given this context, we propose a Team Recruitment Mechanism based on the IPA-TRM. This mechanism transforms the team recruitment problem into the maximum spanning tree problem in graph theory and introduces an improved Prim’s algorithm to seek the optimal solution. To incentivize members to actively participate in collaborative tasks, a team incentive mechanism based on the MR-SVIM was designed. This mechanism comprehensively considers both a member’s direct reputation and indirect reputation. The direct reputation value takes into account the influence of historical reputation values, while the indirect reputation value utilizes the PageRank algorithm to assess a member’s importance within the team. Additionally, a similarity algorithm is employed to compute a member’s weight contribution to the indirect reputation of others. In the profit incentive phase, a negative penalty factor is introduced based on the Shapley value method, ensuring a fair distribution of team benefits based on contribution proportions, guaranteeing each member receives their deserved reward, and avoiding free-riding issues. Finally, experimental validation was conducted to verify the effectiveness and fairness of the IPA-TRM recruitment method and the MR-SVIM profit distribution incentive mechanism. 

## Figures and Tables

**Table 1:** Simulation parameters.

Parameter	Setting
The scale of team *m*	8
Damping factor α	0.85
Controls the shape of the curve ς	1.5
Maximum reputation level *R*	10
Number of teams *T*	8
Total revenue V(M)	500
Total loss gains Loss	50

## Data Availability

Data are contained within the article.
